# Supplementary figures and images for: Viromics unveils extraordinary genetic diversity of the family Closteroviridae in wild citrus
Source: PLoS Pathog. 2021 Jul 12;17(7):e1009751. doi: 10.1371/journal.ppat.1009751 (PMC8297929; doi:10.1371/journal.ppat.1009751)

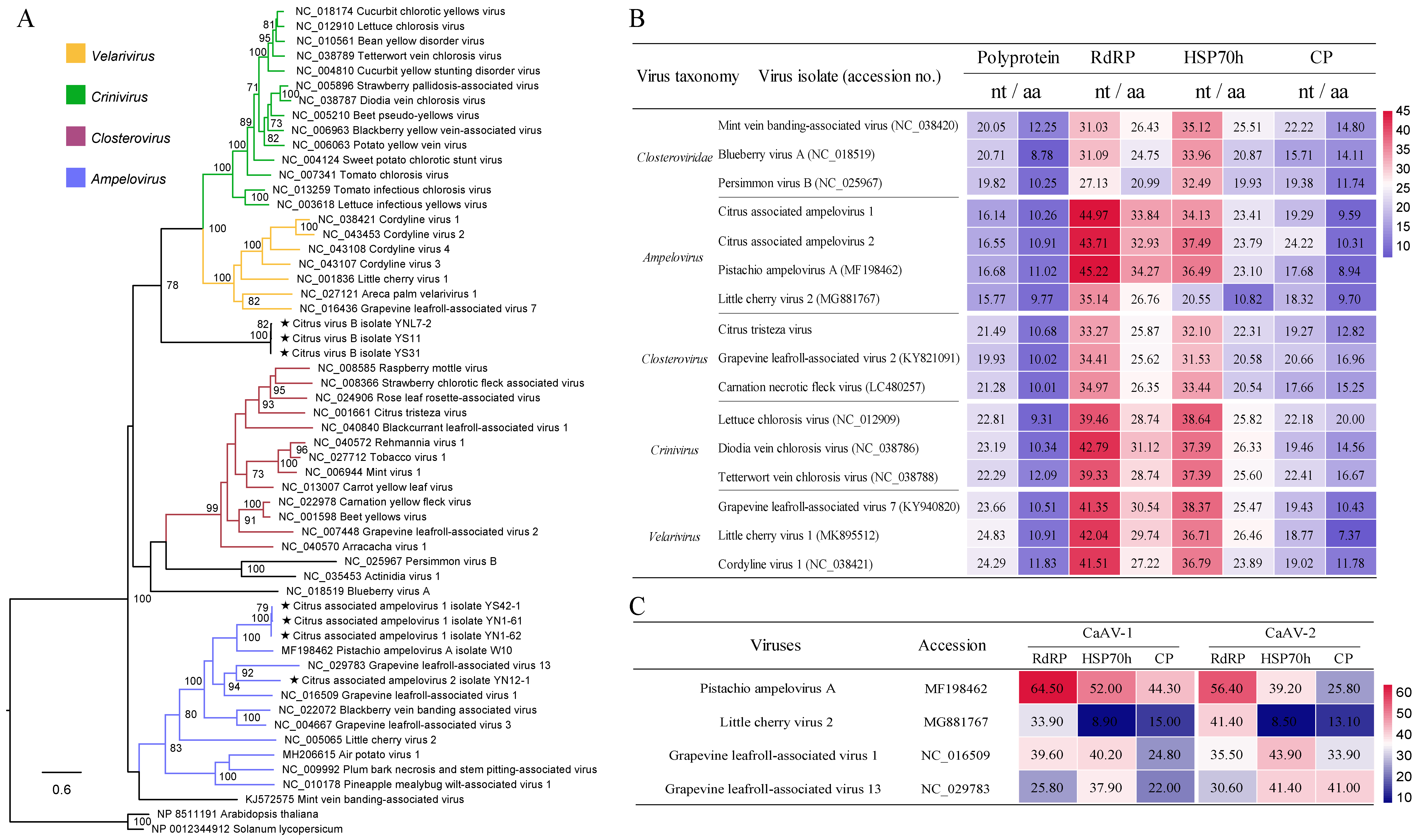

Supplement: S1 Fig — (A) ML tree derived from the HSP70 homolog gene of CiVB, CaAV-1, and CaAV-2, and representative members of the family Closteroviridae. Two plant HSP70 genes were used as an outgroup. Branch support was inferred by bootstrapping with 1,000 replicates. The scale bar represents the number of substitutions per site. Nucleotide and amino acid sequence identities of the different CiVB proteins for the most closely related closterovirids (B) and amino acid sequence identity of CaAV-1 and CaAV-2 proteins for the most closely related ampeloviruses (C). (TIF) [file ppat.1009751.s001.tif]

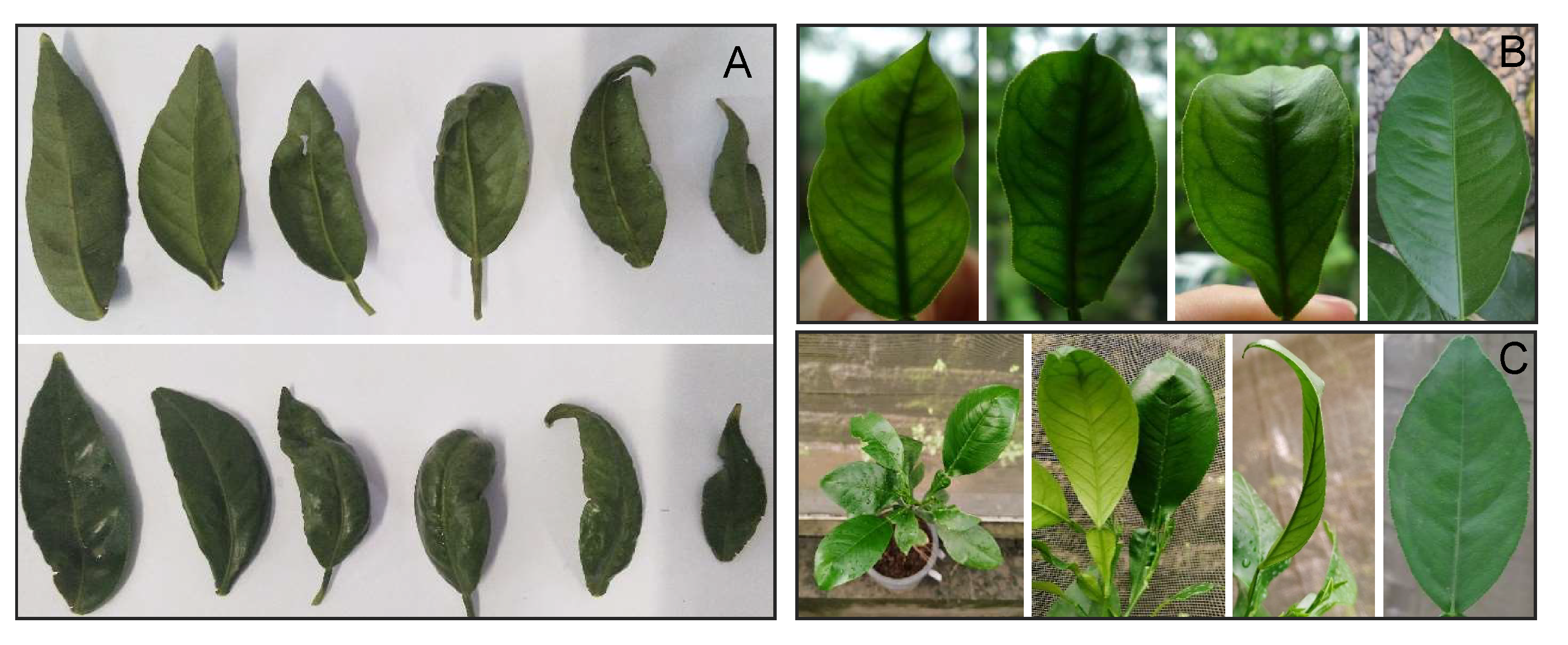

Supplement: S2 Fig — (A) Leafroll symptoms on wild citrus sample YNL8-2. (B) Leaf margin becoming irregular and leaf blade upward or down curling symptoms on Morocco sour orange. (C) Boat-shaped leaf curling symptom on Duncan grapefruit. The right parts of panels B and C represent healthy controls. (TIF) [file ppat.1009751.s002.tif]

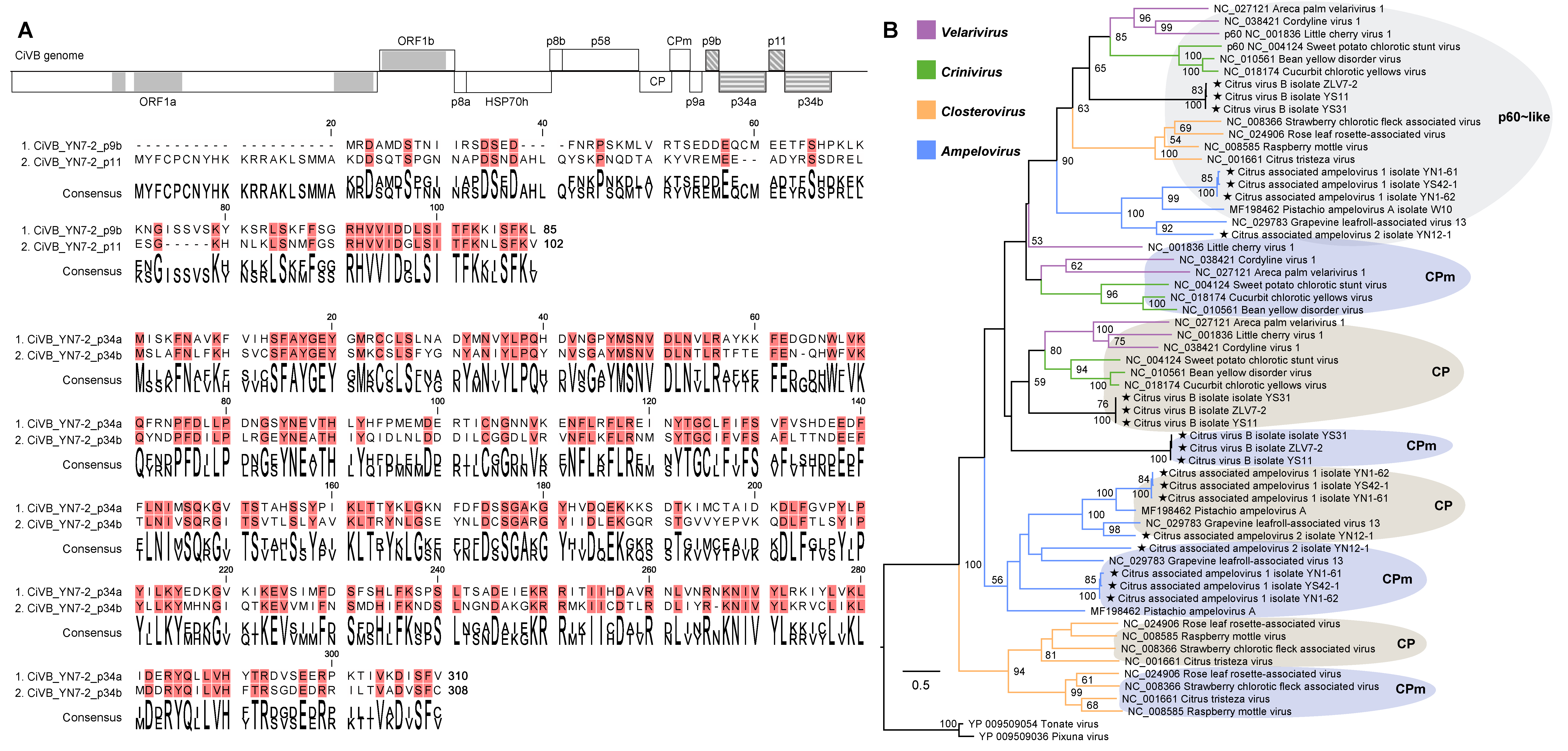

Supplement: S3 Fig — (A) Genomic locations and sequence alignment of p9b and p11, p34a and p34b of CiVB. The replicate genes are labeled in the same stripe shape in the CiVB genome, and the conserved sequences in alignment are labeled in red color. (B) ML tree derived from the CP, CPm, and p60~like genes of CiVB, CaAV-1, CaAV-2, and representative members of the family Closteroviridae. Two alphaviruses were used as an outgroup. Branch support was inferred by bootstrapping with 1,000 replicates. The scale bar represents the number of substitutions per site. (TIF) [file ppat.1009751.s003.tif]

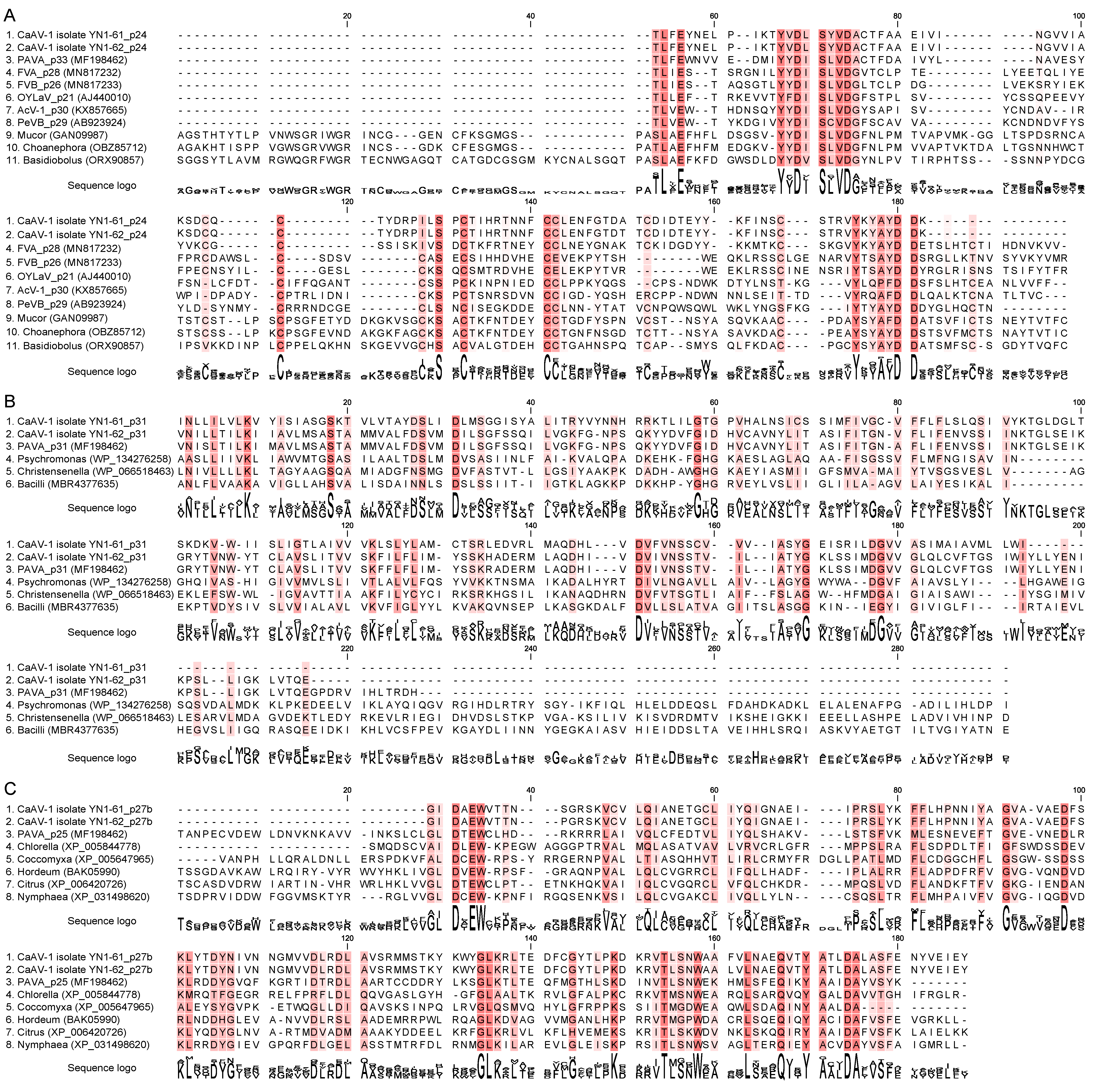

Supplement: S4 Fig — Sequence alignment for closterovirids TLP (A), divalent metal cations transporter (B), and DEDDy 3’-5’ exonuclease (C) genes with their respective homologs. CaAV-1, citrus associated ampelovirus 1; PAVA, pistachio ampelovirus A; FVA, fig virus A; FVB, fig virus B; OYLaV, Olive leaf yellowing-associated virus; PeVB, Persimmon virus B; AcV-1, Actinidia virus 1. (TIF) [file ppat.1009751.s004.tif]

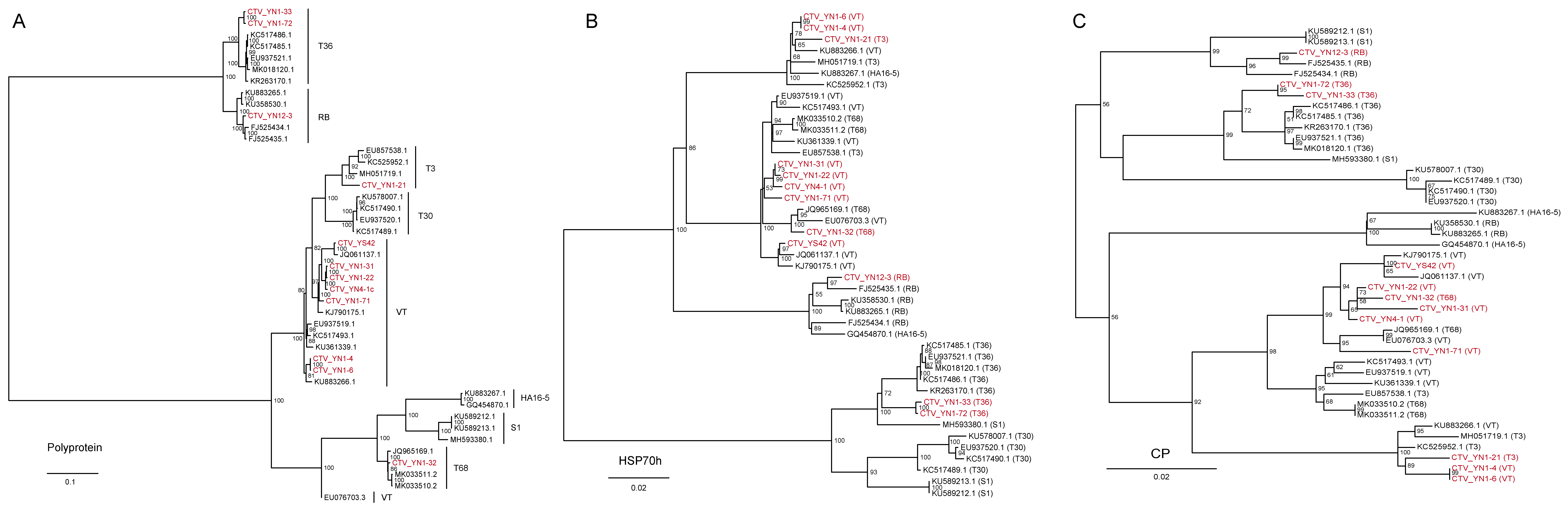

Supplement: S5 Fig — ML trees reconstructed from the nucleotide sequences of the polyprotein (A), HSP70h (B), and CP (C). The values on each node are the percentages of 1,000 bootstrap replicates supporting the branch pattern. Scale bars represent numbers of substitutions per site. CTV isolates from wild citrus are in red. (TIF) [file ppat.1009751.s005.tif]

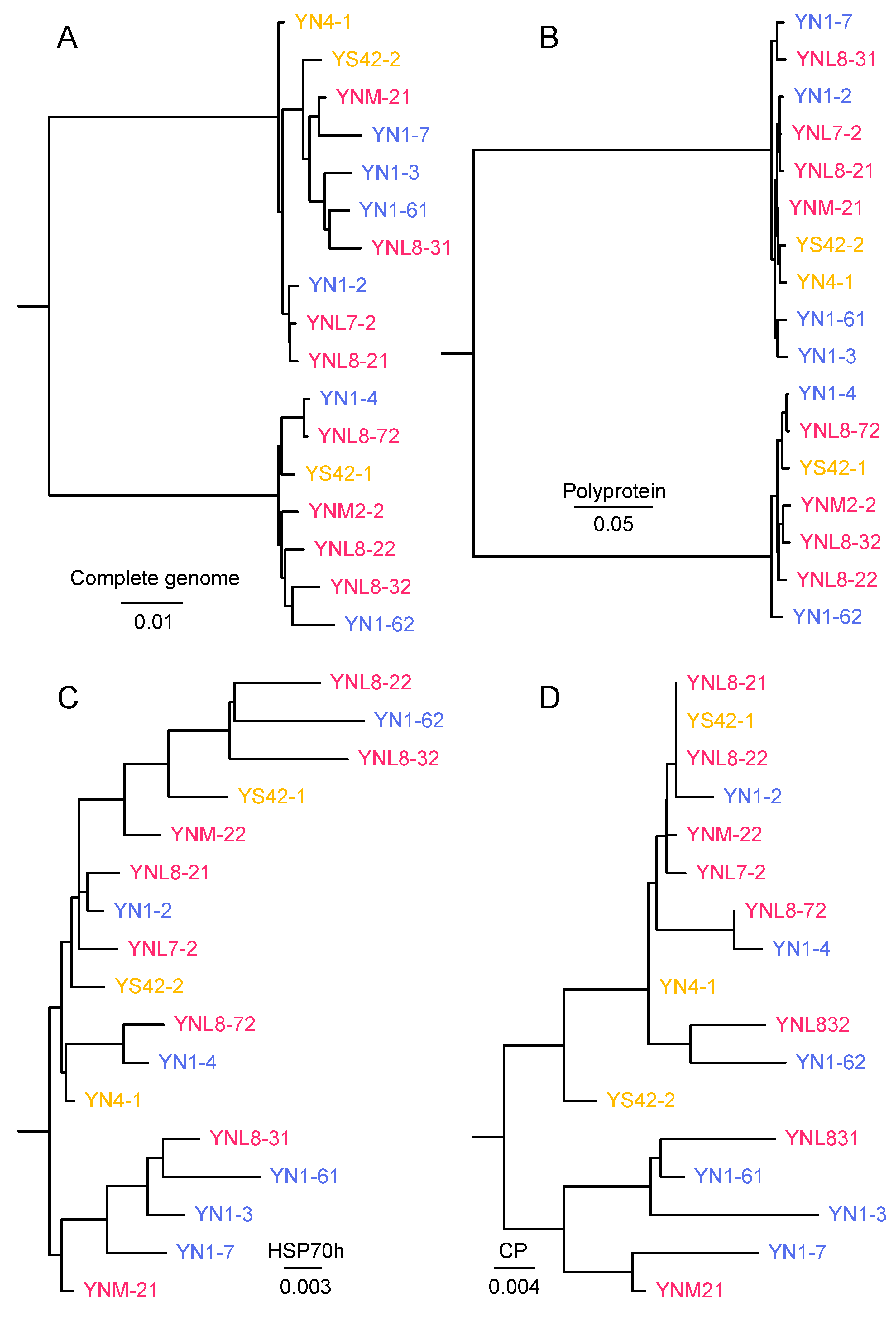

Supplement: S6 Fig — ML trees for CaAV-1 reconstructed from the complete genome sequence (A) and nucleotide sequence of the polyprotein (B), HSP70h (C), and CP (D). The values on each node are the percentages of 1,000 bootstrap replicates supporting the branch pattern. Scale bars represent numbers of substitutions per site. Different colors for clade names represent the origin of the samples: yellow, location 1; pink, location 2; blue, location 3. (TIF) [file ppat.1009751.s006.tif]

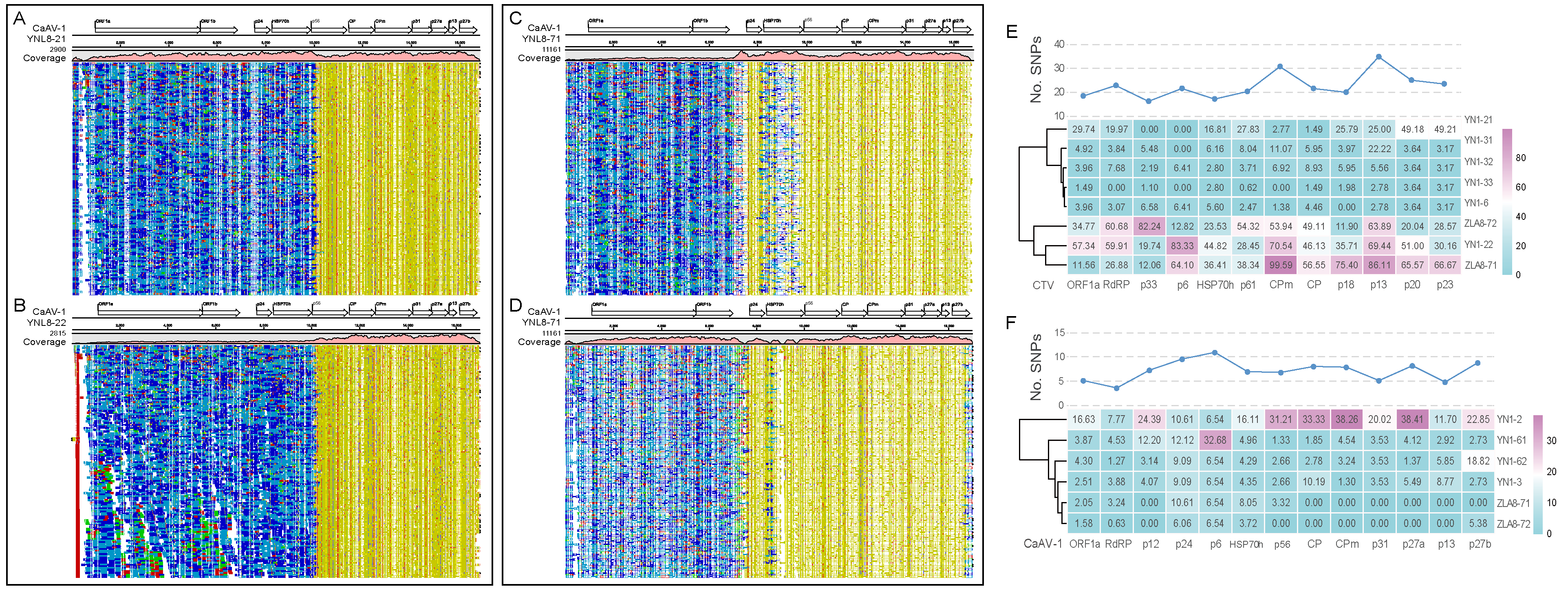

Supplement: S7 Fig — The left part represents two special examples of 3’-half genome recombination of CaAV-1. Transcriptome mapping of CaAV-1 isolates YNL8-21 (A), YNL8-22 (B), YNL8-71 (C), and YNL8-72 (D) identified in samples YNL8-2 (A and B) and YNL8-7 (C and D). The isolates YNL8-22 and YNL8-72 belong to genotype A; YNL8-21 and YNL8-71 belong to genotype B. Shared redundant reads are in yellow. The right part represents sequence variation schemes of CTV (E) and CaAV-1 (F). The levels of sequence variation per 1,000 nt in coding regions of CTV and CaAV-1 isolates are shown in heatmaps; means are shown in the line charts (top). RdRP, RNA-dependent RNA polymerase; HSP70h, heat shock protein 70 homolog; CP, major coat protein; CPm, minor coat protein. (TIF) [file ppat.1009751.s007.tif]
