# Supplementary material for: Viromics unveils extraordinary genetic diversity of the family Closteroviridae in wild citrus
Source: PLoS Pathog. 2021 Jul 12;17(7):e1009751. doi: 10.1371/journal.ppat.1009751 (PMC8297929; doi:10.1371/journal.ppat.1009751)
Supplement: Attachment — Submitted filename: Response to editors and reviewers.docx [file ppat.1009751.s013.docx]

**Response to editors and reviewers**

Dear editors and reviewers,

Thank you for giving us the opportunity to submit a revised draft of our manuscript titled “Viromics unveils extraordinary genetic diversity of the family *Closteroviridae* in wild citrus” to PLOS Pathogens. The comments are all insightful and very helpful for revising and improving our paper, as well as the important guiding significance to our researches. We have studied comments carefully and have made corrections which we hope meet with approval.

On editor’s suggestion, we have invited Guan-Zhu Han and Jianhua Wang who expertise on virus evolution, as coauthors and discussed the research with them extensively. The manuscript has also been revised again by the team of LetPub for correct English usage. The responses to the reviewer’s comments (in blue) can be found in the following pages.

Yours sincerely,

Mengji Cao

May-22-2021

**Part I - Summary**
*Reviewer #1: This work describes discovery of the CTV and three novel closteroviruses in the wild citrus growing in its natural mountainous habitat in China. Given the significant negative impact of CTV on citrus industry worldwide and the potential of new emerging virus infections to affect citrus growers, this work provides important insight into understanding ecology and evolution of citrus-infecting closteroviruses. Moreover, the genomic layout of the new closteroviruses described in this study significantly broadens understanding of genomic and evolutionary plasticity of this rapidly growing virus family and is likely to contribute to revision of closterovirus phylogeny and taxonomy.
In its present form, however, this manuscript needs to be significantly revised and re-written to address a number of the following major comments concerned with the quality of presented analyses and writing.*

**Response:** Thanks for your positive but critical comments on our work and for your suggestions to improve our manuscript further. We have reconfirmed and corrected our analysis on your suggestion. The language of the manuscript has been professionally edited.

*Reviewer #2: This is an innovative paper is providing molecular evidence for previously unrealized cases of infections of wild citrus relatives by two novel virus species belonging to ampelovirus genera and a third virus representing a possible new genus in the Closteroviridae family. Besides extending the citrus virus and the closteroviridae catalogs, this ms shows the significance of natural reserves as potential sources of disease agents endangering cultivated crop plants. Furthermore locating two and possibly three of the Closteroviridae family genera representatives within a limited area of undisturbed natural reserve could also indicate on the close association of the family origin with wild citrus On the other hand the finding of 5 out of the seven main CTV strains reported throughout the citrus world, associated with these wild citrus relatives either indicates , that CTV also originated in this area or that the native vegetation became infested by CTV strains carried by long distance flying vectors derived from long distance cultivated citrus hosting these isolates. For completing this paper the authors are suggested to include a supplemental map indicating on the distances of commercially cultivated citrus in the surrounding areas of the reserve.*

**Response:** Thanks for your good suggestions. We supplemented a map here indicating the wild citrus sampling locations and surrounding commercially cultivated citrus (Fig.1). Only in recent years have these citrus trees been planted. We would screen these plants furtherly in the future to explore the virus ecology in wild and cultivated citrus.

Fig.1 Locations of wild citrus sampling and surrounding commercially cultivated citrus.

**Part II – Major Issues**
*Reviewer #1:*

*1. The study would significantly benefit from providing data on: i) virus incidence (% of the wild plants infected with each of the 4 viruses); ii) occurrence of the mixed infections;*

**Response:** We have supplemented the virus incidence and mixed infections of different viruses in the “Discovery of closterovirids in wild citrus” section of the Results as well as in Fig 1D, E and S3 Table. In addition, mixed infections of different genotypes of citrus tristeza virus (CTV) and citrus associated ampelovirus 1 (CaAV-1) have also been supplemented in the “Genotyping, recombination, and asymmetrical genomic variation of CTV and CaAV-1 in wild citrus” section of the Results. We listed the corresponding results below.

“In a total of 49 wild citrus samples, the incidence of CaAV-1 (14/49, 28.6%) was similar to CTV (16/49, 32.7%), both higher than CiVB (9/49, 18.4%). CaAV-2 was only detected in one sample in location 4 (Fig 1D). Mixed infection was detected in 16 of 49 samples (32.7%), in which all CiVB isolates were mixed with CaAV-1, CTV or both (S3 Table). ”

“From the viromics analysis, mixed infection of different genotypes existing within a single sample as higher in CaAV-1 (8/14, 57.1%) than in CTV (4/10, 40%).”

*iii) presence of disease symptoms in the screened wild plants and, most importantly, in the cultivated Morocco orange plants used for grafting. Although not strictly required, addition of such data would significantly expand the biological/plant pathological dimension of this work, which, at this point, is entirely focused on virus phylogenomics.*

**Response:** The symptoms of citrus virus B (CiVB) were not included in the analysis as all CiVB isolates were mixed infected with CaAV-1 or CTV. The Morocco sour orange with CaAV-1 and CaAV-2 detecting positive has been grafted on different citrus seedlings to detect their biological features. However, the time has not long enough that we have only detected CaAV-1 positive on Duncan grapefruit up to now.

Biological symptoms of CaAV-1 were accordingly added. The symptoms of wild citrus with single infected of CaAV-1 as well as the grafted Morocco sour orange and Duncan grapefruit were described in results of ”Discovery of closterovirids in wild citrus” and S2 Fig. Corresponding changes are listed below.

“Among the collected wild citrus samples, the sample YN8-2, which was only infected by CaAV-1 without any other viruses or viroids, exhibited leafroll symptoms, as shown in S2A Fig. As all CiVB isolates showed mixed infection with other citrus closterovirids, it was difficult to analyze their biological symptoms. After eleven months of grafting, three Morocco sour orange [*Citrus. aurantium*] plants inoculated with YN8-2 and that were CaAV-1 positive exhibited a series of symptoms (S2B Fig), with the leaf margin becoming irregular and the leaf blade upward or down curling. The barks of CaAV-1 positive sour orange was subsequently grafted on a Duncan grapefruit (*C*. *paradisi*) which exhibits boat-shaped leaf curling (S2C Fig) after five months of grafting.”

The Morocco sour orange with CaAV-2 infection has not shown abnormal symptoms comparing to the healthy control. However, we detected CaAV-2 in three pomelos (*Citrus. maxima*) which were collected in Guangxi Province and introduced from Thailand. The pomelo samples were used to construct an rRNA-depleted RNA library that was then sequenced on the X-ten platform. That confirmed the pomelos are single infected by CaAV-2. The samples exhibit a severe leafroll and yellowing symptom as shown below (Fig.2). But the pomelo trees were removed by farmers later that we can not investigate them further. Further studying the biological features of these novel citrus closterovirids is the focus of our next work.

Fig.2 the symptom of pomelo that single infected by CaAV-2.

*2. By and large, the data presentation and interpretation shows insufficient expertise of the authors in molecular biology of the closteroviruses and virus evolution in general. But a few telling examples are as follows: i) In Fig. 3, line 162 and elsewhere, the closterovirus ~60 KDa proteins are named ‘HSP90h’ (for homologs of HSP90 cellular molecular chaperones), a long obsolete notion refuted in the work by Napuli et al. (2003; PMID:12551975); ii) terms ‘intraspecies’ or ‘external genes’ are not in use in the field;*

**Response:** We have read the references carefully and corrected these misstatements accordingly. In addition, we have significantly revised the manuscript.

*iii) claiming that CiVB with monopartite genome is likely an ancestor of the criniviruses with split genomes is a run-away speculation that has no support in the data (in RdRP tree – Fig. 4 – CiVB is as divergent from criniviruses as it goes and so is CiVB CP in the same figure);*

**Response:** We have reanalyzed the phylogenetic relationship of CiVB and the results are in concordance with your opinion (CiVB is as divergent from criniviruses as it goes and so is CiVB CP in the same figure). Thus, we deleted corresponding results and discussions.

*iv) statement that some of the closterovirus genes that are not universally conserved in the family are under negative selection (e.g., lines 319-321) has no basis in presented data: gene gain and loss is one of the dominant processes in genome evolution that cannot possibly be branded as positive or negative selection; etc., etc.*

**Response:** For point IV, this is a misstatement that has been deleted and the result and discussion of gene gain and loss has been rewritten. We have reanalyzed and discussed about the results.

*3. The reliability of the conserved domain identification in this study is a suspect. In the lines 171-172, it is casually mentioned that CaAV-1 genome encodes two subunits of the D**NA polymerase III: none of the RNA viruses known so far does encode any part of any DNA polymerase (there is no DNA phase in their replication cycles). If this is correct, such finding must be supported by identifying such domains in more than one isolate of this virus as well as presenting reliable, statistically robust multiple alignment of these subunits from CaAV-1 and diverse cellular organisms. Same is required for ‘divalent metal cations transport protein’ and ‘DEDDy 3’-5’ exonuclease (lines 176-178). If correct, the latter finding would be the only exonuclease encoded by RNA viruses in addition to those in coronaviruses, potentially a very significantly discovery suggestive of the proof-reading capability of this virus.*

**Response:** We have reconfirmed the conserved domain searching results with new standards that only genomic features identified in all isolates and E-value < 1E-5 are considered reliable.

As the DNA polymerase III was only identified in one of the genotypes of CaAV-1 with a low confidence level, we deleted this result. The genes that encode the thaumatin-like protein, divalent metal cations transport protein, and DEDDy 3’-5’ exonuclease have been reconfirmed to exist in all virus isolates. the E-value of the genomic features has been added. We supplemented the multiple sequence alignment (S4 Fig) and phylogenetic analysis of these genes (Fig 5).

*Further, the lack of L-Pro in all three newly discovered viruses would be exceedingly surprising (if true) and more likely hints at rather low sensitivity of the employed search software.*

**Response:** We identified L-Pro in CiVB genomes using manual sequence alignment (Fig.2) and labeled its genomic location. However, we did not identify L-Pro in CaAV-1 and CaAV-2 genomes. We noticed that all viruses that clustered with CaAV-1 and CaAV-2 (pistachio ampelovirus A and little cherry virus 2) in the rdrp tree (Fig 2 in manuscript) lack an L-Pro domain. And considering the fixed locations of L-Pro in closterovirids, the lack of L-Pro in CaAV-1 and CaAV-2 genomes should be reliable since their methyltransferase domain very close to their N-termini of open reading frame (ORF) 1a. (We also tried to identify L-Pro in central variable regions of ORF1a of CaAV-1 and CaAV-2 but failed.)

Fig.2 Multiple sequence alignment of L-Pro of CiVB and other closterovirids.

LIYV, lettuce infectious yellows virus; DVCV, Diodia vein chlorosis virus; MVBaV, mint vein banding-associated virus; BYV, beet yellows virus.

*4. The quality of language and writing in general are also significant problems. But a few beyond the pale examples are sentences in lines 18-23, 291, 336-338.*

**Response:** The manuscript has been revised again by the team of LetPub to ensuring correct usage of language. The enumerated instances above have been deleted.

*The entire Discussion is excessively long and largely incoherent;*

**Response:** We have reorganized the entire Results and Discussion of the manuscript. The horizontal gene transfer and new results of gene duplication were integrated into a new section of “Formation of genomic complexity of the family Closteroviridae” in Results. The sections of “Intraspecies of CTV and CaAV-1 in wild citrus plants” and “How recombination and variation affect asymmetrically divergent genomes” have been integrated into a new section of “Genotyping, recombination, and asymmetrical genomic variation of CTV and CaAV-1 in wild citrus”.

*The section ‘Unique mode…’ starting on line 236 belongs to Discussion in much briefer form (experimental confirmation of the lack of frameshift should be mentioned), many acronyms in the text were never spelled out etc., etc.*

**Response:** The ‘Unique mode…’ section has been deleted and corresponding results were briefly mentioned in the revised Discussion (lines 349-356). We have revised the manuscript carefully to avoid the mistakes of the reviewer enumerated.
